# Mammary gland growth and vascularity at parturition and during lactation in primiparous ewes fed differing levels of selenium and nutritional plane during gestation

**DOI:** 10.1186/2049-1891-4-6

**Published:** 2013-02-26

**Authors:** Tammi L Neville, Allison M Meyer, Arshi Reyaz, Pawel B Borowicz, Dale A Redmer, Lawrence P Reynolds, Joel S Caton, Kimberly A Vonnahme

**Affiliations:** 1 Center for Nutrition and Pregnancy, Department of Animal Sciences, North Dakota State University, Fargo 58108, USA

**Keywords:** Mammary gland, Nutrition, Proliferation, Selenium, Vascularity

## Abstract

**Background:**

Objectives were to examine the effects of selenium (Se) supply and maternal nutritional plane during gestation on mammary gland growth, cellular proliferation, and vascularity at parturition and d 20 of lactation. Rambouillet primiparous ewes (n = 84) were allocated to treatments in a 2 x 3 factorial. Factors were dietary Se (adequate Se [**ASe**, 11.5 μg/kg BW] or high Se [**HSe**, 77.0 μg/kg BW]) and nutritional plane (60% [**RES**], 100% [**CON**], or 140% [**EXC**]). At parturition, lambs were removed and 42 ewes (7/treatment) were necropsied. Remaining ewes were fed a common diet meeting requirements for lactation and mechanically milked twice daily until necropsy on d 20. At both necropsy periods, mammary glands were dissected and tissues harvested. Samples were analyzed for RNA, DNA, and protein content, cell proliferation, and vascularity. Where interactions were present (*P* ≤ 0.05), least squares means from the highest-order interaction are presented.

**Results:**

Final body weight of ewes was least (*P* ≤ 0.002) in RES, intermediate for CON, and greatest for EXC, regardless of stage of the ewe at necropsy (parturition or d 20 of lactation). In ewes necropsied at parturition, mammary glands were heavier (*P* = 0.02) in EXC compared to RES, with CON intermediate. Concentration of RNA (mg/g) was decreased (*P* = 0.01) in EXC compared to CON at parturition. There was a tendency (*P* = 0.07) for a Se by nutrition interaction in percentage of cells proliferating where ASe-EXC ewes had greater (*P* ≤ 0.02) number of proliferating cells then all other treatments. Mammary vascular area tended (*P* = 0.08) to be affected by a Se by nutrition interaction where ASe-CON had less (*P* = 0.007) vascular area than HSe-CON ewes. In ewes necropsied at d 20 of lactation, the number of alveoli per area was decreased (*P* ≤ 0.05) in RES compared to CON and EXC-fed ewes.

**Conclusions:**

Results of this study indicate that proper maternal nutritional plane during gestation is important for mammary gland development, even out to d 20 of lactation.

## Background

Maternal nutrition directly influences milk quantity and quality available to offspring [1,2]. Previously, Swanson et al. [3] reported decreased colostrum production in primiparous ewes due to maternal under- and over-nutrition during pregnancy, which corresponded with a decreased mammary gland weight in the undernourished ewes. Furthermore, these authors [3] report decreased mammary cellular proliferation in the alveoli, and increased alveolar area, in undernourished primiparous ewes. Ewes that were restricted during mid to late gestation had decreased milk production compared to control and overfed ewes, even when fed similar levels of nutrients for the first 20 d of lactation [2]. Meyer et al. [2] also demonstrated that supranutritional selenium (Se) during gestation may enhance milk production in ewes. Mammary gland growth, milk yield, and mammary tissue DNA content was influenced by energy and protein intake in sows during lactation [4]. Moreover, increased dietary lipids in peripubertal ewe lambs resulted in increased mammogenesis [5].

Development of the mammary gland in ewes from birth through puberty, gestation, and into d 5 of lactation was outlined by Anderson [6], where the author concluded that unlike many species, sheep do not exhibit post-parturient growth of the mammary gland (i.e., during lactation). Our laboratory has recently demonstrated that capillary density of mammary alveoli at parturition is increased in ewes that received high Se throughout gestation, whereas impacts of differing nutritional plane during gestation were less dramatic [7].

We hypothesized that Se supplementation during gestation would increase vascular density at different periods of mammary growth. Moreover, we hypothesized that under- and over- nutrition during pregnancy would negatively affect mammary gland growth, development, and vascularity. Therefore, the objectives of this study were to determine how maternal Se supplementation and nutritional plane during gestation influence mammary tissue growth, cellular proliferation, and vascularity and if realimentation to a common diet during lactation can reverse the effects.

## Materials and methods

### Animals and diets

This experiment was approved by the Institutional Animal Care and Use Committee at North Dakota State University. Ewes were bred and managed as described in Meyer et al. [2,8]. Breeding occurred at the U.S. Sheep Experiment Station, at this time, Se treatments [adequate Se (**ASe**; 3.5  g Se kg/BW•d) or high Se (**HS**e; 65  g Se kg/BW•d)] were initiated. After shipping to North Dakota State University at d 36 of gestation, pregnant Rambouillet primiparous ewes (n = 84; 52.1 ± 6.2 kg) were individually housed. Ewes remained on their Se treatments (actual intakes: ASe, 11.5 μg Se kg⁄BW•d; HSe, 77.0 μg Se kg⁄BW•d), and on d 40 of gestation were assigned randomly to 1 of 3 nutritional plane treatments supplying 60% (**RES**), 100% (**CON**), or 140% (**EXC**) of NRC (1985) recommendations for 60 kg pregnant ewe lambs during mid to late gestation (weighted ADG of 140 g) except for Se. This resulted in a completely randomized design with a 2 × 3 factorial of Se supply × nutritional plane (ASe-RES, ASe-CON, ASe-EXC, HSe-RES, HSe-CON, and HSe-EXC).

All diets were fed once daily in a complete pelleted form and based on wheat middlings, beet pulp, alfalfa meal, and ground corn. Three pellet formulations (basal, high Se, and concentrated Se pellets; described in [8]) were blended to meet Se and metabolizable energy (**ME**) intake based upon the Se treatment and nutritional plane of each individually penned ewe. The basal pellet contained 15.9% crude protein (**CP**) and 2.81 Mcal/kg ME [dry matter (**DM**) basis]. Selenium sources used were Se-enriched wheat mill run to replace wheat middlings and corn in the basal diet to make a high Se pellet (6.13 ppm Se, 16.6% CP, 2.82 Mcal/kg ME; DM basis) and purified seleno-methionine added to achieve 37.1 ppm Se in the concentrated Se pellet (16.2% CP, 3.01 Mcal/kg ME; DM basis). Every 14 d, body weight (**BW**) was measured and diets were adjusted accordingly.

### Parturition and lactation

All births were attended and lambs were removed immediately for artificial rearing. Ewes assigned to necropsy on d 20 of lactation were transitioned over 5 d to a diet providing 100% of NRC [9] requirements for early lactation, delivered by a combination of the basal pellet fed during gestation and a protein pellet (50.2% CP and 2.6 Mcal ME/kg; DM basis; soybean meal, wheat middlings, urea, and mineral supplement) and fed in 2 portions, 1 after each milking. Ewes were mechanically milked twice a day [2] at 0500 and 1700 until necropsy which occurred after the 0500 milking on d 20. As we have previously reported [2], there were main effects of both Se supply and nutritional plane on colostrum and milk yield. Colostrum and milk yields were greater in HSe ewes compared to ASe ewes. Additionally, ewes fed CON plane of nutrition had greater colostrum weight and volume than RES and EXC [2]. Milk production from the EXC ewes increased on the second day of lactation to meet levels of milk yield from CON ewes. Both EXC and CON ewes had greater milk production than RES ewes [2].

### Slaughter procedures

Ewes assigned to necropsy at parturition and lactation were slaughtered 3 to 24 h post-partum or after the 0500 milking and feeding on d 20 of lactation, respectively. Immediately before slaughter, ewes were weighed and mammary glands were stripped of residual milk accumulated since the last mechanical milking by manual milking after treatment with 1 mL of oxytocin (20 IU; AgriLabs, St. Joseph, MO) to facilitate milk ejection. Animals were stunned by captive bolt (Supercash Mark 2; Accles and Shelvoke Ltd., Sutton Coldfield, UK), exsanguinated, and detailed necropsies performed. The mammary gland was dissected from the skin, weighed, and processed. From one half of the mammary gland, 5 samples (approximately 1 g each) of glandular tissue were snap frozen in super-cooled isopentane (submerged in liquid nitrogen) and stored at −80°C until analysis for RNA, DNA, and protein. The remaining half of the mammary gland was perfusion fixed with Carnoy’s fixative (60% ethanol, 30% chloroform, 10% glacial acetic acid) by cannulating the cranial mammary artery with a polyethylene (PE-60; o.d. = 1.22 mm; i.d. = 0.77 mm; Intramedic, Becton Dickinson & Company, Sparks, MD) beveled catheter that was secured to surrounding tissue. The mammary gland was initially perfused with PBS, then Evan’s blue dye (to define the perfusion area), and then PBS again, and finally, was perfusion fixed with Carnoy’s fixative. Perfused tissue was cut into 5 mm thick slices and was further immersion fixed in Carnoy’s fixative for an additional 8 h and then moved to 70% alcohol for storage. Thereafter, mammary gland tissues were dehydrated in a series of ethanol and Histo-clear (National Diagnostics, Atlanta, GA) rinses and embedded in paraffin wax.

### Cellularity estimates

Freshly thawed tissue samples were homogenized using a Polytron with a tPT-10s probe (Brinkman, Westbury, NY) in Tris aminomethane, sodium, and EDTA buffer (TNE buffer; 0.05 mol/L Tris, 2.0 mol/L NaCl, 2 m mol/L EDTA, pH 7.4). Samples were analyzed for concentrations of DNA and RNA using the diphenylamine [10] and orcinol procedures [11], respectively. Protein concentrations in tissue homogenates were determined with Coomassie brilliant blue G [12], with bovine serum albumin (Fraction V; Sigma, St. Louis, MO) as the standard [10]. Prepared samples were analyzed with a spectrophotometer (Beckman DU640, Beckman Coulter Inc., Fullerton, CA) and were assessed against concentration curves of known standards. Concentration of DNA was used as an index of hyperplasia, and RNA:DNA and protein:DNA ratios were used as an index of hypertrophy and potential cellular activity [13,14].

### Cellularity and vascularity

Paraffin-embedded tissues were sectioned at 4 μm, and stained for a cellular proliferation marker using the mouse anti-proliferating nuclear cell antigen (PCNA) primary antibody (Chemicon International, Temecula, CA) and detected with a biotinylated secondary antibody (horse anti-mouse IgG, Vectastain; Vector Laboratories, Burlingame, CA) and the Avidin-Biotin Complex system (Vectastain; Vector Laboratories). Tissues were further stained with periodic-acid Schiff’s reagent and counterstained with hematoxylin. Photomicrographs were taken at 400× magnification using a Nikon Eclipse E800 microscope equipped with Nikon DXM 1200 F digital camera (n = 10 pictures per slide, 85,734.7 μm^2^ per picture). Images were analyzed for proliferating alveolar cells and alveolar luminal area, and cellular proliferation was quantified using the Image-Pro Plus 5.0 analysis software (Media Cybernetics, Silver Spring, MD).

**Figure 1:**
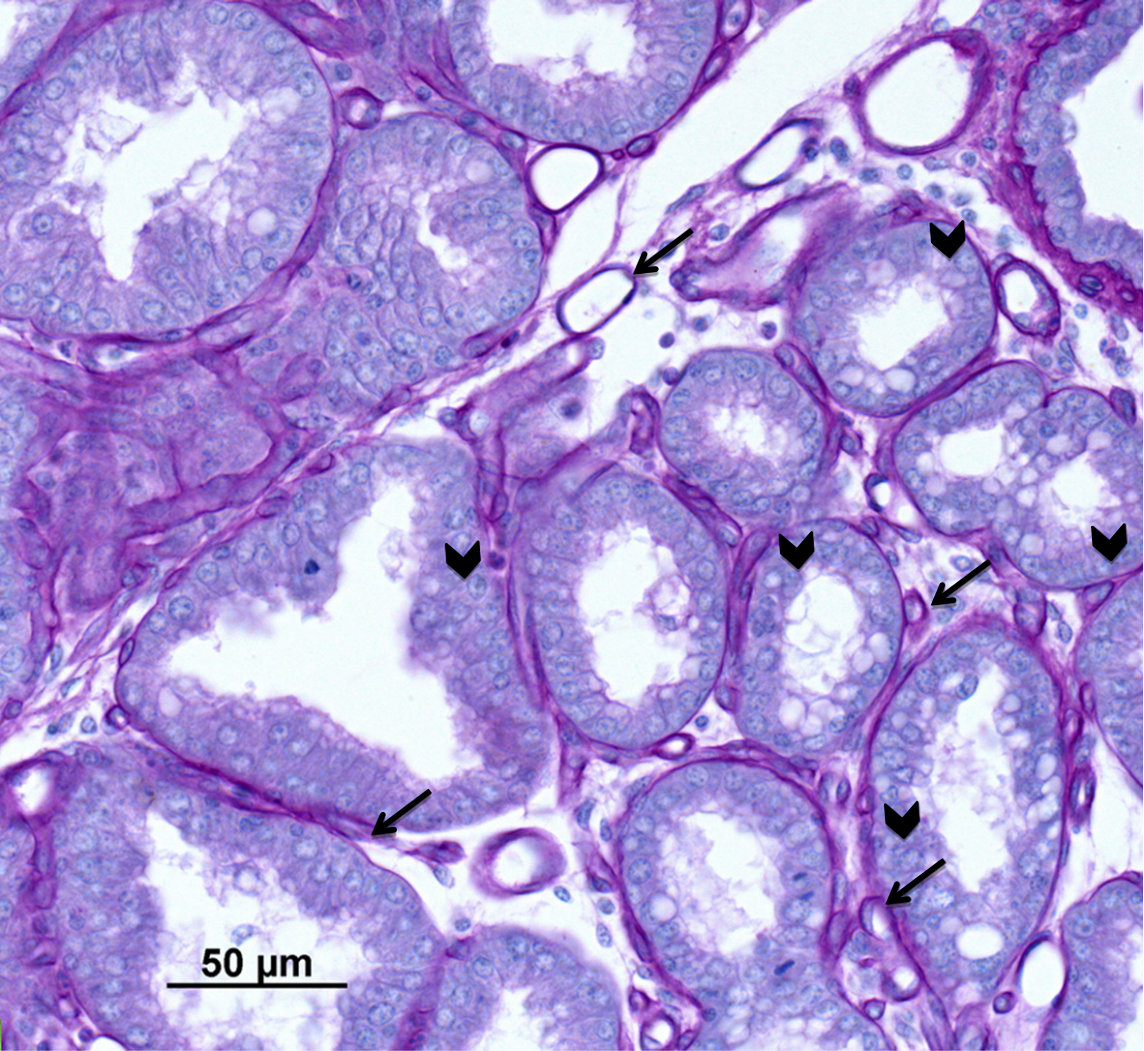
Representative photomicrograph of hematoxylin and periodic acid Schiff’s stained mammary gland tissue with blood vessels (arrows) and alveoli (arrowheads) present.

Vascularity staining was performed using an antibody to Factor VIII, a specific endothelial cell marker, as previously described by our laboratory [15] and counter-stained with using periodic acid-Schiff’s staining procedures to provide contrast to the vascular tissue, as previously described [7,16,17]; Figure 1. Photomicrographs were takes as described above. Vascularity was then determined by image analysis (Image-Pro Plus, version 5.0, Media Cybernetics, Houston, TX). Briefly, for each ewe, 10 images per mammary gland (in the alveolar area) were analyzed for tissue area, luminal area, alveoli, and total vascular area (i.e. area stained for Factor VIII; capillary area per total tissue area).

### Calculations

Empty BW was considered to be digesta weight subtracted from the final BW before slaughter. Digesta weight was calculated by difference (total full viscera weight – visceral tissues after stripping of digesta contents). Total tissue DNA, RNA, and protein contents were calculated by multiplying DNA, RNA, and protein concentration by fresh tissue weights [3,13,18]. Percentage of vascular area was calculated as 100×[((total picture area – luminal area) – (total vascular area))/total vascular area]. Alveoli per glandular area analyzed was calculated as (the number of alveoli/total picture area) × 1,000.

### Statistics

Data were analyzed as a completely randomized design with a 2 × 3 factorial arrangement using GLM procedures of SAS (SAS Inst. Inc., Cary, NC) within each necropsy period (parturition or lactation). The model contained effects for Se (ASe vs. HSe), nutritional plane (RES, CON, and EXC), and their interaction. Means were separated using least significant difference when *P* < 0.05 and tendencies discussed when *P* > 0.05 and < 0.10. In the absence of interactions (*P* > 0.10), main effects are reported (*P* < 0.10); otherwise interactive means are discussed.

## Results

### Parturition

There was a main effect (Table 1; *P* < 0.001) of nutritional plane on final and empty BW where both were least (*P* ≤ 0.003) in RES, intermediate in CON, and greatest (*P* < 0.001) in EXC. Mammary gland weight was affected (*P* = 0.05) by nutritional plane during gestation where RES was less than (*P* = 0.02) EXC. Furthermore, mammary glands from CON tended (*P* = 0.07) to be less than EXC, while there was no difference (*P* = 0.47) between RES and CON. When expressed proportional to empty BW, there were no differences (*P* > 0.13) in mammary gland weight.

**Table 1:** Effects of gestational Se supply and nutritional plane on mammary gland weight, cellularity, cell proliferation, and vascularity when necropsied after parturition

	**Se supply**^**1**^		**Nutritional plane**^**2**^				***P*-value**^**3**^			
**Item**	**ASe**	**HSe**	**SEM**^**4**^	**RES**	**CON**	**EXC**	**SEM**^**5**^	**Se**	**Nut**	**Se×Nut**
Final BW, kg	54.5	54.9	1.4	46.3^a^	54.0^b^	63.8^c^	1.7	0.86	<0.001	0.79
Empty BW^6^, kg	47.8	47.9	1.3	40.2^a^	47.2^b^	56.2^c^	1.7	0.96	<0.001	0.87
Mammary gland, g	754.1	850.7	46.9	715.3^a^	773.5^ab^	918.4^b^	59.6	0.15	0.05	0.55
g/kg EBW^7^	15.9	18.2	1.1	18.0	16.7	16.4	1.4	0.14	0.69	0.33
DNA, mg/g	3.59	3.59	0.24	3.63	3.62	3.53	0.30	0.98	0.96	0.94
DNA, g	2.67	3.00	0.22	2.58	2.79	3.14	0.28	0.30	0.36	0.48
RNA, mg/g	5.49	5.42	0.38	5.60^ab^	6.21^a^	4.56^b^	0.48	0.90	0.04	0.90
RNA, g	3.89	4.55	0.30	3.92	4.57	4.17	0.39	0.13	0.46	0.78
RNA:DNA	1.57	1.52	0.09	1.57^ab^	1.71^a^	1.36^b^	0.11	0.67	0.07	0.41
Protein, mg/g	29.37	25.64	2.64	25.02	27.72	29.79	3.36	0.32	0.59	0.25
Protein, g	22.75	21.54	2.27	18.16^a^	21.16^ab^	27.12^b^	2.89	0.71	0.09	0.10
Protein:DNA	8.80	7.98	1.03	7.86	7.76	9.54	1.32	0.57	0.53	0.51
Proliferation, %	3.38	2.14	0.40	1.63	2.96	3.69	0.51	0.02	0.03	0.07
ASe	--	--	--	1.82^y^	3.06^y^	5.27^z^	0.75			
HSe	--	--	--	1.44^y^	2.86^y^	2.11^y^	0.68			
Vascular area, %	48.74	51.27	1.08	50.78	49.47	49.77	1.35	0.09	0.76	0.33
Alveoli per area^7^	0.57	0.53	0.02	0.52	0.55	0.59	0.02	0.23	0.10	0.46

^a,b,c^ Within an item, main effect means differ (*P* ≤ 0.05).

^y,z^ Within an item, interactive means differ (*P* ≤ 0.02).

^1^Ewes fed 11.5 μg/kg BW Se (ASe) or 77.0 μg/kg BW Se (HSe) during gestation.

^2^Ewes fed 60% (RES), 100% (CON), or 140% (EXC) of nutrient requirements from d 40 of gestation to term.

^3^Probabilities of difference for Se supply (Se), nutritional plane (Nut), and the interaction (Se×Nut).

^4^Standard error means for ASe n = 19 and HSe n = 19.

^5^Standard error means for RES n = 12, CON n = 14, and EXC n = 12.

^6^Empty BW (EBW) = Final BW – digesta weight.

^7^Alveoli per area = (number of alveoli/total picture area) ×1,000.

There were no differences (Table 1; *P* > 0.29) in mammary gland DNA (mg/g or g) due to Se, nutrition, or their interaction. Concentration of RNA (mg/g) was altered (*P* = 0.04) due to nutritional plane where CON was greater (*P* = 0.01) than EXC and RES was intermediate. When total RNA was calculated there were no differences (*P* > 0.12) due to Se, nutrition, or their interaction. There was a tendency (*P* = 0.07) for a main effect of nutrition on RNA:DNA where EXC was reduced (*P* = 0.02) when compared to CON with RES similar (*P* > 0.17) to both. Total protein (g) tended (*P* = 0.09) to be affected by nutritional plane with EXC having greater (*P* = 0.03) protein compared to RES and CON being intermediate. There were no differences (*P* > 0.24) due to Se, nutrition, or their interaction for protein concentration (mg/g) or protein:DNA.

Percentage of proliferating cells within the mammary gland tended (Table 1; *P* = 0.07) to be affected by a Se by nutrition interaction where ASe-EXC was greater (*P* ≤ 0.02) than all other treatments. Vascular area tended (*P* = 0.09) to be greater in HSe ewes compared to ASe. Alveoli per area were similar (*P* ≥ 0.10) across dietary treatments.

### Lactation

There was a main effect (Table 2; *P* < 0.001) of gestational nutritional plane on final and empty BW where both were least (*P* ≤ 0.001) in RES, intermediate in CON, and greatest (*P* ≤ 0.001) in EXC. There were no effects (*P* > 0.12) on mammary gland weight (g or g/kg empty BW) due to Se, nutrition, or their interaction.

**Table 2:** Effects of gestational Se supply and nutritional plane on mammary gland weight, cellularity, cell proliferation, and vascularity when necropsied after d 20 of lactation

	**Se supply**^**1**^		**Nutritional plane**^**2**^				***P*-value**^**3**^			
**Item**	**ASe**	**HSe**	**SEM**^**4**^	**RES**	**CON**	**EXC**	**SEM**^**5**^	**Se**	**Nut**	**Se×Nut**
Final BW, kg	55.9	57.3	1.3	48.5^a^	56.7^b^	64.6^c^	1.6	0.46	<0.001	0.56
Empty BW^6^, kg	45.9	47.73	1.25	39.1^a^	46.9^b^	54.4^c^	1.6	0.29	<0.001	0.31
Mammary gland, g	577.7	634.4	31.7	541.8	628.1	648.4	38.8	0.21	0.13	0.74
g/kg EBW^7^	12.6	13.5	0.8	14.0	13.4	11.7	0.9	0.42	0.19	0.78
DNA, mg/g	3.43	3.26	0.12	3.15	3.48	3.40	0.14	0.30	0.26	0.89
DNA, g	1.98	2.10	0.15	1.71^a^	2.23^b^	2.18^b^	0.18	0.54	0.09	0.96
RNA, mg/g	8.50	8.10	0.39	7.46	8.76	8.68	0.48	0.48	0.11	0.37
RNA, g	5.00	5.22	0.43	4.14^a^	5.59^b^	5.60^b^	0.52	0.71	0.09	0.69
RNA:DNA	2.49	2.49	0.09	2.39	2.52	2.56	0.11	0.99	0.53	0.13
Protein, mg/g	27.71	30.83	2.62	28.19	30.40	29.23	3.21	0.41	0.89	0.48
Protein, g	16.20	19.77	2.05	15.37	19.33	19.25	2.51	0.22	0.45	0.74
Protein:DNA	8.26	9.55	0.87	9.18	8.93	8.60	1.07	0.30	0.93	0.50
Proliferation, %	9.81	11.15	1.34	10.55	11.11	9.78	1.64	0.85	0.48	0.62
Vascular area, %	45.37	48.59	1.36	48.20	46.75	46.00	1.69	0.10	0.64	0.08
ASe	--	--	--	48.01^y^	42.07^z^	46.03^yz^	2.48			
HSe	--	--	--	48.38^y^	51.42^y^	45.96^yz^	2.29			
Alveoli per area^7^	0.54	0.52	0.03	0.45^a^	0.55^b^	0.59^b^	0.03	0.45	0.02	0.33

^a,b,c^ Within an item, main effect means differ (*P* ≤ 0.07).

^y,z^ Within an item, interactive means differ (*P* ≤ 0.09).

^1^Ewes fed 11.5 μg/kg BW Se (ASe) or 77.0 μg/kg BW Se (HSe) during gestation.

^2^Ewes fed 60% (RES), 100% (CON), or 140% (EXC) of nutrient requirements from d 40 of gestation to term.

^3^Probabilities of difference for Se supply (Se), nutritional plane (Nut), and the interaction (Se×Nut).

^4^Standard error means for ASe n = 21 and HSe n = 21.

^5^Standard error means for RES n = 14, CON n = 14, and EXC n = 14.

^6^Empty BW (EBW) = Final BW – digesta weight.

^7^Alveoli per area = (number of alveoli/total picture area) ×1,000.

Total DNA (g) tended (Table 2; *P* = 0.09) to be affected by nutritional plane where less (*P* = 0.05) DNA was present in mammary glands from RES compared to CON with EXC intermediate. There were no differences (*P* > 0.25) in DNA concentration (mg/g) due to Se, nutrition, or their interaction. There was a tendency (*P* = 0.09) for nutritional plane to affect total RNA (g) where RES had decreased (*P* = 0.05) RNA compared to EXC, with CON intermediate. There were no differences (*P* > 0.10) due to Se, nutrition, or the interaction on RNA concentration (mg/g), RNA:DNA, protein (mg/g and g), or protein:DNA.

Proliferating cells were not affected (Table 2; *P* > 0.47) by gestational Se, nutrition, or their interaction. There was a tendency (*P* = 0.08) for a Se by nutrition interaction on vascular area. Vascularity in HSe ewes did not differ regardless of nutritional plane. However, in ASe ewes, RES ewes had greater vascular area compared to CON ewes with EXC intermediate. Moreover, HSe-CON ewes had increased (*P* = 0.007) vascular area compared to ASe-CON. Alveoli per area was affected (*P* = 0.02) by nutritional plane where RES was decreased (*P* ≤ 0.05) compared to CON and EXC.

## Discussion

The ovine mammary gland appears to be quite dynamic in its ability to compensate from differing gestational diets to common lactational diets within a 20 d period. In terms of mammary gland growth and vascularity there were four key observations when comparing measures from parturition to lactation. First, while mammary gland weight was associated with level of dietary intake at parturition, after 20 d of a common lactational diet, there were no differences in mammary gland weight. Second, the enhanced proliferation in ASe-EXC alveoli at parturition was no longer observed after 20 d of lactation, although proliferation was greatly enhance from parturition to lactation. Third, while there was only a tendency for Se to increase mammary gland vascularity at parturition, by d 20 of lactation, it appears that ewes fed HSe-CON diets during gestation had enhanced vascularity of the gland compared to those fed ASe-CON ewes. Lastly, while the number of alveoli per area at parturition was not impacted by maternal gestational diet, by d 20 of lactation, RES ewes had fewer alveoli compared to CON and EXC ewes.

The decreased mammary gland weight observed at parturition for under- compared with over-fed ewes is similar to results published by Swanson et al. [3] with these authors additionally noting a decreased weight in under-fed compared with controls. In the current study mammary gland weight tended to be less in CON vs. EXC, while we previously reported [3] mammary glands of control and over-fed ewes were of similar weight. Others have reported ewes consuming ad libitum diets had greater mammary gland weights than ewes consuming a maintenance diet [19]. Mammary gland growth was also impaired within 3 d of late gestation nutrient restriction where decreased gland mass was recorded at parturition [20] even when ewes were realimented during the last 5 d of pregnancy [21].

Total DNA measured in the mammary glands of the current study was reduced in under- compared to over-fed ewes at 20 d of lactation in the current study. Previously, Swanson et al. [3] reported DNA of under-fed ewes was reduced compared to control ewes at parturition, whereas in the current study no differences were found at parturition. Anderson [6] has reported DNA concentration was greatest in ewes near term, with no differences in total DNA between the end of pregnancy and d 5 of lactation. Our results for total RNA at 20 d of lactation are similar to those of Swanson et al. [3] at parturition, where both showed a reduction in RNA of under-fed compared to control ewes. Additionally, these authors [3] found under-fed ewes to have reduced total RNA compared to over-fed ewes. Anderson [6] reported similar RNA concentrations between end of pregnancy and early lactation, but greater total RNA at 5 d of lactation.

The greater proliferating cells in the mammary glands of over-fed ewes that were also fed adequate Se compared to all others at parturition is similar to results of Swanson et al. [3], where there was an increase in proliferation in over- compared to under-fed ewes. While colostrum production was reduced in ewes that were overnourished [2,3], overnourished ewes can catch up in milk yield within with first few days of lactation [2]. Perhaps the enhanced proliferation of the alveoli that was noted in this study assists in increasing milk yield. Moreover, while there was no difference in the number of alveoli at parturition, RES ewes had reduced alveolar numbers compared to CON and EXC at 20 d of lactation. Perhaps this can explain why previously restricted ewes during gestation could not achieve milk production yields within 20 d of lactation, even though offered similar dietary nutritional intake [2]. It is of interest to determine how maternal nutritional plane could impact the endocrine profile of ewes to further explain the altered milk production in these ewes.

Current vascular area data are supported by previous research where, at parturition, capillary vascularity was greatly enhanced in high-Se fed ewes [7]. Our laboratory has published enhanced milk yields from HSe vs. ASe ewes [2]. Perhaps the ability of the mammary gland to increase in vascularity (current study) by d 20 of lactation assisted with this increased yield.

The mammary gland is a very dynamic organ that is influenced by gestational nutrition, and it appears that while overnourished ewes can compensate from poor colostrum yields to adequate milk production, ewes that are restricted may not be able to achieve this [2]. The mechanism for compensatory milk yield may lie in the relationship between dietary changes and enhanced proliferation and vascularity of the mammary gland.

## Competing interests

The authors declare no competing interest regarding the content or conclusions expressed in this research.

## Authors’ contributions

The experimental design was conceived by JSC, DAR, LPR, and KAV. Animal portion of the experiment was executed by TLN, AMM, DAR, LPR, JSC, and KAV. Histology work was performed by PPB and AR. Data analyses were performed by TLN, AMM, JSC, and KAV. TLN and KAV were the primary authors of the paper, but all authors participated in the final version of the paper. All authors read and approved the final manuscript.
